# Increased Circulating Endothelial Apoptotic Microparticle to Endothelial Progenitor Cell Ratio Is Associated with Subsequent Decline in Glomerular Filtration Rate in Hypertensive Patients

**DOI:** 10.1371/journal.pone.0068644

**Published:** 2013-07-12

**Authors:** Chien-Yi Hsu, Po-Hsun Huang, Chia-Hung Chiang, Hsin-Bang Leu, Chin-Chou Huang, Jaw-Wen Chen, Shing-Jong Lin

**Affiliations:** 1 Division of Cardiology, Department of Medicine, Taipei Veterans General Hospital, Taipei, Taiwan; 2 Department of Medical Research and Education, Taipei Veterans General Hospital, Taipei, Taiwan; 3 Healthcare and Management Center, Taipei Veterans General Hospital, Taipei, Taiwan; 4 Institute of Clinical Medicine, National Yang-Ming University, Taipei, Taiwan; 5 Cardiovascular Research Center, National Yang-Ming University, Taipei, Taiwan; 6 Institute and Department of Pharmacology, National Yang-Ming University, Taipei, Taiwan; University of Padova, Medical School, Italy

## Abstract

**Background:**

Recent research indicates hypertensive patients with microalbuminuria have decreased endothelial progenitor cells (EPCs) and increased levels of endothelial apoptotic microparticles (EMP). However, whether these changes are related to a subsequent decline in glomerular filtration rate (GFR) remains unclear.

**Methods and Results:**

We enrolled totally 100 hypertensive out-patients with eGFR ≥30 mL/min/1.73 m^2^. The mean annual rate of GFR decline (△GFR/y) was −1.49±3.26 mL/min/1.73 m^2^ per year during the follow-up period (34±6 months). Flow cytometry was used to assess circulating EPC (CD34^+^/KDR^+^) and EMP levels (CD31^+^/annexin V^+^) in peripheral blood. The △GFR/y was correlated with the EMP to EPC ratio (*r* = −0.465, *p*<0.001), microalbuminuria (*r* = −0.329, *p* = 0.001), and the Framingham risk score (*r* = −0.245, *p* = 0.013). When we divided the patients into 4 groups according to the EMP to EPC ratio, there was an association between the EMP to EPC ratio and the ΔGFR/y (mean ΔGFR/y: 0.08±3.04 vs. −0.50±2.84 vs. −1.25±2.49 vs. −4.42±2.82, *p*<0.001). Multivariate analysis indicated that increased EMP to EPC ratio is an independent predictor of ΔeGFR/y.

**Conclusions:**

An increased circulating EMP to EPC ratio is associated with subsequent decline in GFR in hypertensive patients, which suggests endothelial damage with reduced vascular repair capacity may contribute to further deterioration of renal function in patients with hypertension.

## Introduction

It has been recognized that declining renal function, estimated by the glomerular filtration rate (GFR), is an independent risk factor for all-cause mortality [1], [2] and poorer outcomes in cardiovascular disease (CVD) [2]–[4]. Clinical studies in hypertensive patients and patients at high cardiovascular risk have shown that classic risk factors such as diabetes mellitus, hyperlipidemia, and smoking, are major correlates of renal dysfunction [5]–[7]. Since clinical evidence indicates that declining renal function is associated with an increased risk for cardiovascular and all-cause mortality, identification of hypertensive individuals at high risk for developing chronic kidney disease (CKD) is an important issue for primary and secondary prevention of CKD in the community.

It has become evident that the vascular endothelium is a highly active organ that affects vascular tone, smooth muscle cell proliferation, monocyte adhesion, and platelet aggregation [8]. Endothelial dysfunction has been shown to play a critical role in the clinical manifestations of established atherosclerotic lesions [9], [10]. Of note, previous reports have demonstrated that endothelial dysfunction is present in the early stages of renal insufficiency, and that it is associated with a greater decline in renal function [11], [12]. Recent evidence suggests that injured endothelial monolayer is regenerated partly by bone marrow derived-endothelial progenitor cells (EPCs), and circulating EPC level was shown to be associated with endothelial function [13]. Reduced EPC numbers independently predict progression of atherosclerotic disease and future cardiovascular events, which suggests a pivotal role of endogenous vascular repair by EPCs in modulation of the clinical course of coronary artery disease (CAD) [14]–[18]. Endothelial microparticles carry membrane proteins and phospholipids of the parent cell and can be derived from microparticles of leukocytes, erythrocytes, or platelets [19]. Endothelial apoptotic microparticle (EMP) levels are elevated in conditions of endothelial cell damage, and accumulating evidence also suggests that in addition to being a consequence of vascular damage, endothelial microparticles may have a proatherogenic role [20]–[23]. Our recent study showed that hypertensive patients with nephropathy have increased EMP levels and decreased numbers of circulating EPCs [24]. However, information is lacking about the possible contribution of circulating EMPs and EPCs to the progression of renal disease. Thus, we designed this study to prospectively evaluate the role of EMPs and EPCs in renal function decline in a group of well-characterized hypertensive patients.

## Methods

### Study Participants

From April 2008 through December 2008, we consecutively recruited 100 outpatients with essential hypertension and a baseline estimated GFR ≥30 mL/min/1.73 m^2^. Hypertension was defined as a systolic blood pressure ≥140 mmHg, a diastolic blood pressure ≥90 mmHg, or use of antihypertensive drugs. Subjects with history or clinical evidence of angina, myocardial infarction, congestive heart failure, peripheral vascular disease, inflammatory disease, or any disease predisposing to vasculitis were excluded. Causes of secondary hypertension were excluded by appropriate investigations. Patients with stage 4 and 5 chronic kidney disease (GFR <30 mL/min/1.73 m^2^) were also excluded [25]. During a 4-year follow-up period to April 2012, 100 of patients (62 men and 38 women; mean age 62±14 years) were available for analysis with a mean follow-up duration of 34±6 months. The study was approved by the research ethics committee of Taipei Veterans General Hospital (Taipei Veterans General Hospital Institutional Review Board, No: 96-12-42A), and all participants provided their written informed consent.

### Clinical Evaluation

Medical history, including cardiovascular risk factors, previous and present cardiovascular events, and current medication regimen, was obtained during a personal interview and from medical files. All the measurements were made at the medical center after an overnight fast of at least 8 hours. Weight, height, and waist circumference were measured and body mass index (BMI) was calculated. Brachial blood pressure was measured by a physician with a mercury sphygmomanometer after patients sat for 15 minutes or longer. The average of 3 measurements was used for the analysis.

### Measurement of Renal Function

Creatinine measurements were performed at baseline and at the end of follow-up using the Jaffe method implemented in an auto-analyzer. Estimated GFR values (eGFR, mL/min/1.73 m^2^) were calculated with the new equation proposed by investigators in the Chronic Kidney Disease Epidemiology (CKD-EPI) Collaboration [26]. This equation was developed from a cohort of patients that included both healthy individuals and individuals with chronic kidney disease that was much larger than the Modification of Diet in Renal Disease (MDRD) study. We prefer this equation because it is more accurate in subjects with eGFR >60 mL/min/1.73 m^2^, and it has been externally validated in Chinese populations with greater precision and accuracy in terms of eGFR [27], [28]. Reassessment of renal function was undertaken in all patients (range 24–48 months), and the annual rate of decline of eGFR (ΔeGFRy) was determined by subtraction the eGFR value at reassessment from the eGFR at baseline divided by the time interval in years between the 2 evaluation periods for each patient.

### Laboratory Measurements

Venous blood samples were collected from all patients after 8 hours of overnight fasting for measurement. The blood samples were centrifuged at 3000 rpm for 10 minutes immediately after collection, and the plasma samples were kept frozen at −70°C until analysis. The hsCRP level was determined using a latex-enhanced immunonephelometric assay (Dade Behring, Marburg, Germany). Plasma NT-proBNP was determined by a sandwich immunoassay (EIMA) with two antibodies (Cortez Diagnostics, Calabasas, CA, USA). Each standard and plasma sample was analyzed twice, and the mean value was used in all subsequent analyses. The intra-assay and inter-assay variation coefficients of the tests were <10% [29].

### Assay of Circulating CD31^+^/Annexin V^+^ Apoptotic Microparticles

**Figure 1:**
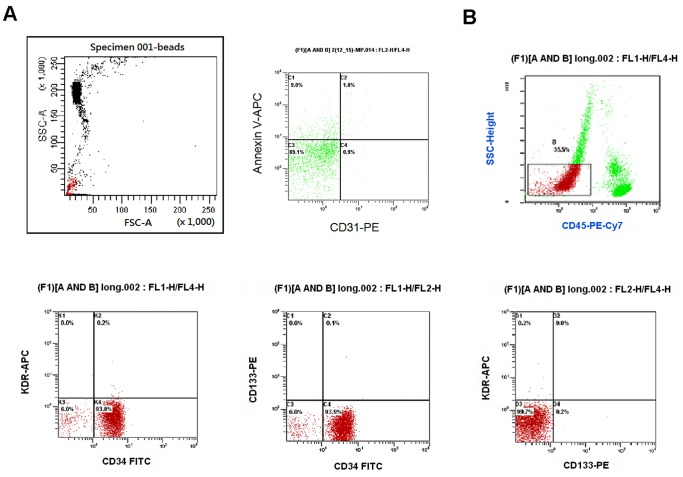
Representative flow cytometry analysis for quantifying the number of endothelial progenitor cells and endothelial microparticles. (A) The gate was set using sizing microparticles (<1.5mm). Endothelial apoptotic microparticles were defined as particles positively labeled for CD31 and annexin V (CD31^+^/annexin V^+^). (B) Mononuclear cells (MNCs) were gated by forward/sideward scatter (FSC/SSC). The numbers of circulating endothelial progenitor cells (EPCs) were gated with monocytes and defined as CD34^+^KDR^+^CD133^+^. FITC, fluorescein isothiocyanate.

Plasma derived from 10 mL citrate-buffered blood was immediately centrifuged at 13,000 *g* for 2 minutes to generate platelet-poor plasma. Fifty microliters of platelet-poor plasma was incubated with 4 μL of phycoerythrin (PE)-conjugated monoclonal antibody against CD31 (Becton Dickinson) followed by incubation with fluorescein isothiocyanate (FITC)-conjugated annexin V according to the manufacturer’s instructions. IgG-FITC (Pharmingen, San Jose, California, USA) served as the negative control. Fluorescence-activated cell sorting (FACS) analysis was performed immediately after staining using a FACSCalibur flow cytometer (Becton Dickinson). We used standard beads (Nile Red fluorescent particles, 1.7–2.2 micro meter, catalog number 556261, BD) to identify microparticles in gating cells by FACS. CD31^+^/annexin V^+^ microparticles were defined as particles positively labeled for CD31 and annexin V (CD31^+^/annexin V^+^). Representative flow cytometry analysis for quantifying the number of endothelial apoptotic microparticles is shown in Figure 1A. To reduce the number of microparticles derived from non-endothelial cells, which may occasionally show low expression of CD31^bright^, only CD31^+^ microparticles were selected [24]. This approach does not allow exclusive measurement of endothelial cell-derived microparticles, but may imply that platelet-derived microparticles are additionally measured. However, previous study showed an acceptable correlation between levels of CD31^+^/annexin V^+^ and CD31^+^/CD42^−^ microparticles in 104 patients (r = 0.89, P<0.001) [30], [31]. Data were analyzed using Cellquest software (Becton Dickinson).

### Assay of Circulating Endothelial Progenitor Cells

Assessment of the circulating EPCs by flow cytometry was performed by the researchers masked to the clinical data. A volume of 1000-µL peripheral blood was incubated for 30 minutes in the dark with monoclonal antibodies against human kinase insert domain-conjugating receptor (KDR; R&D, Minneapolis, Minnesota, USA) followed by PE-conjugated secondary antibody, with the fluorescein isothiocyanate (FITC)-labeled monoclonal antibodies against human CD45 (Becton Dickinson, Franklin Lakes, New Jersey, USA), and PE-conjugated monoclonal antibody against human CD133 (Miltenyi Biotec, Bergisch Gladbach, Germany), and with FITC-conjugated or PE-conjugated monoclonal antibodies against human CD34 (Serotec, Raleigh, North Carolina, USA) and KDR (Sigma, St Louis, Missouri, USA). Isotype-identical antibodies served as controls (Becton Dickinson). After incubation, cells were lysed, washed with phosphate-buffered saline (PBS), and fixed in 2% paraformaldehyde before analysis. Each analysis included 100,000 events. As shown in Figure 1B, the numbers of circulating EPCs were gated with monocytes and defined as CD34^+^KDR^+^CD133^+^. To assess the reproducibility of EPC measurements, circulating EPCs were measured from 2 separate blood samples in 10 subjects, and there was a strong correlation between the two measurements (r = 0.88, *p*<0.001) [29], [32].

### Statistical Analysis

The analysis was performed on the complete data set, and results were expressed as mean ± SD or as percent frequency. Comparisons between two groups were made by paired or unpaired Student *t* test, Mann-Whitney *U* test, or Chi square test, as appropriate. Comparisons of continuous variables among the 4 groups were performed by analysis of variance (ANOVA). Subgroup comparisons of categorical variables were assessed by Chi square or Fisher’s exact test. Variables associated with the annual rate of decline of eGFR were identified using Pearson’s correlation coefficient or Spearman’s correlation coefficient, as appropriate. Linear regression analysis was used as appropriate to assess the relationship between annual rate of decline of eGFR, traditional risk factors, medications, and CD31^+^/annexin V^+^ EMP to EPC ratio. Finally, we constructed multivariable models using △eGFR as the dependent coefficient. Regression model results are presented as correlation coefficients and their *p* values. Data were analyzed using SPSS software (version 20, SPSS, Chicago, Illinois, USA). A *p* value of less than 0.05 was considered to indicate statistical significance.

## Results

### Patient Characteristics

The mean age of the 100 hypertensive patients (62 males, 62%) was 62±14 years. The baseline characteristics of the patients are listed in Table 1. All study subjects were evaluated periodically for clinical, biochemical, and cardiovascular measurements. Renal function was determined in all study subjects at baseline and at 34±6 months later (range 24–48 months). The median duration of follow-up for all patients in the trial was 34 months (interquartile range, 31 to 38). The baseline mean eGFR was 82.44±20.92 mL/min/1.73 m^2^, which decreased to 78.34±23.51 mL/min·1.73 m^−2^ by the end of the observation period. In the general population, the normal annual mean decline in GFR with age from the peak GFR (120 mL/min·1.73 m^−2^) attained during the third decade of life is 1 mL/min/1.73 m^2^ per year, reaching a mean value of 70 mL/min/1.73 m^2^ by age 70 [32]. In our study cohort, the mean yearly decline in GFR was 1.49±3.26 mL/min/1.73 m^2^ per year, which was similar with the natural history of CKD and was not statistically different between males and females (*p = *0.230).

**Table 1:** Baseline characteristics of study population.

	All Subjects (n = 100)
Age (years)	62±14
Male	62 (62%)
Diabetes	14 (14%)
BMI	25.9±3.2
Current smoker	36 (36%)
Lipid profile	
Total Cholesterol (mg/dL)	193±37
Triglycerides (mg/dL)	163±120
HDL (mg/dL)	44±9
LDL (mg/dL)	116±34
Fasting glucose (mg/dL)	112±33
Serum Cr (mg/dL)	0.96±0.33
Uric acid (mg/dL)	6.2±1.5
Initial eGFR (mL/min·1.73 m^−2^)	82.44±20.92
Follow-up eGFR (mL/min·1.73 ^−^m^2^)	78.34±23.51
Mean follow-up duration (months)	34±6
Mean △eGFR/y (mL/min·1.73 m^−2^)	−1.49±3.26
Systolic BP (mmHg)	142±20
Diastolic BP (mmHg)	81±14
FRS (%)	9.5±7.6
MAU (ACR)	0.07±0.18
Medications	
ACE-I	15 (15%)
ARB	69 (69%)
CCB	76 (76%)
Beta-blocker	32 (32%)
Diuretics	32 (32%)
Statin	21 (21%)
Antihypertensive therapyduration (years)	7.7±6.2
EMP (n/μL)	259.6±377.6
EPC (n/mL)	63.1±60.1
EMP/EPC ratio	20.8±70.8

Values are mean ± SD or number (%).

BMI, body mass index; HDL, high-density lipoprotein; LDL, low-density lipoprotein; Cr, creatinine; eGFR, estimated glomerular filtration rate; △eGFR/y, annual rate of change of eGFR; BP, blood pressure; FRS, Framingham risk score; MAU, microalbuminuria; ACR, albumin/creatinine ratio; ACE-I, angiotensin-converting enzyme inhibitor; ARB, angiotensin II receptor blocker; CCB, calcium channel blocker; EMP, endothelial apoptotic microparticle; EPC, endothelial progenitor cell.

### Circulating EMP to EPC Ratio in Hypertensive Patients with Subsequent Deterioration of Renal Function

Based on the EMP/EPC ratio, the actual cutoff values for each quartile of the EMP/EPC ratio are 0.5, 2.0, and 11.7. We classified our patients into four groups according to quartiles, including 25 patients (25%) in group 1 (Q1) with EMP/EPC ratio ≤0.5, 25 patients (25%) in group 2 (Q2) with EMP/EPC ratio >0.5 and ≤2.0, 25 patients (25%) in group 3 (Q3) with EMP/EPC ratio >2.0 and ≤11.7, and 25 patients (25%) in group 4 (Q4) with EMP/EPC ratio >11.7. The baseline characteristics of the patients in each group are presented in Table 2. There were no significant differences among the 4 groups with respect to age, sex, diabetes, smoking status, serum levels of total cholesterol, HDL-cholesterol, LDL-cholesterol, triglycerides, baseline serum creatinine, systolic blood pressure, diastolic blood pressure, uric acid, initial eGFR, or Framingham risk score. However, patients in group 3 had a significantly higher body mass index, and there was increasing level of microalbuminuria (ACR, albumin/creatinine ratio) among the 4 groups (*p = *0.028). There were no significant differences in their antihypertensive therapy duration and medication usage among the 4 groups, including angiotensin converting enzyme inhibitors, angiotensin II receptor blockers, calcium-channel blockers, beta-blockers, statins, and thiazides.

**Table 2:** Baseline characteristics in 4 groups of hypertensive patients according to EMP/EPC ratio.

EMP/EPC ratio	Q1n = 25	Q2n = 25	Q3n = 25	Q4n = 25	*p* value
Age (yrs)	63±12	63±12	63±16	62±14	0.988
Men	16 (64%)	17 (68%)	12 (48%)	17 (68%)	0.439
Diabetes	1 (4%)	2 (8%)	6 (24%)	5 (20%)	0.112
BMI	24.7±3.3	25.3±2.7	27.3±3.3	26.2±3.2	0.025
Current smoker	12 (48%)	8 (32%)	8 (32%)	8 (32%)	0.612
Lipid profile					
T. Chol	189±31	198±34	184±44	199±36	0.400
Triglyceride	143±79	146±91	192±175	163±120	0.412
HDL	44±10	46±9	44±9	41±9	0.247
LDL	116±27	122±29	102±42	123±36	0.093
Fasting glucose	103±14	115±41	118±40	110±28	0.387
Serum Cr	0.88±0.19	0.93±0.27	0.98±0.39	1.02±0.41	0.452
Uric acid	6.0±1.2	6.2±1.5	6.3±1.9	6.4±1.4	0.848
Initial eGFR	87.0±18.0	83.2±16.0	79.3±24.7	80.2±24.3	0.555
Systolic BP	137±15	141±23	148±22	143±18	0.253
Diastolic BP	79±10	83±17	82±15	80±13	0.649
FRS	10.0±7.4	8.3±6.2	8.7±7.6	11.2±9.1	0.508
MAU (ACR)	0.01±0.01	0.05±0.10	0.07±0.14	0.16±0.31	0.028
hsCRP	0.30±0.27	0.38±0.49	0.38±0.31	0.29±0.23	0.659
NT-pro-BNP	92.2±32.7	92.4±71.2	88.8±40.3	70.6±50.1	0.382
Medications					
ACE-I	7 (28%)	3 (12%)	2 (8%)	3 (12%)	0.227
ARB	15 (60%)	16 (64%)	20 (80%)	18 (72%)	0.311
CCB	18 (72%)	20 (80%)	18 (72%)	20 (80%)	0.816
Beta-blocker	9 (36%)	9 (36%)	6 (24%)	8 (32%)	0.828
Thiazides	6 (24%)	8 (32%)	11 (44%)	7 (28%)	0.113
Statin	6 (24%)	4 (16%)	6 (24%)	5 (20%)	0.870
Antihypertensive therapy duration(years)	7.6±6.3	6.2±5.5	9.2±6.7	7.8±6.2	0.375

Values are mean ± SD or number (%).

EMP, endothelial apoptotic microparticle; EPC, endothelial progenitor cell; BMI, body mass index; T. Chol, total cholesterol (mg/dL); HDL, high-density lipoprotein (mg/dL); LDL, low-density lipoprotein (mg/dL); Cr, creatinine (mg/dL); eGFR, estimated glomerular filtration rate (mL/min/1.73 m^2^/year); BP, blood pressure (mmHg); FRS, Framingham risk score (%); MAU, microalbuminuria; ACR, albumin/creatinine ratio; hs-CRP, high-sensitivity C-reactive protein (mg/dL); T-pro-BNP, N terminal pro- brain natriuretic peptide (pg/mL); ACE-I, angiotensin-converting enzyme inhibitor; ARB, angiotensin II receptor blocker; CCB, calcium channel blocker.

**Figure 2:**
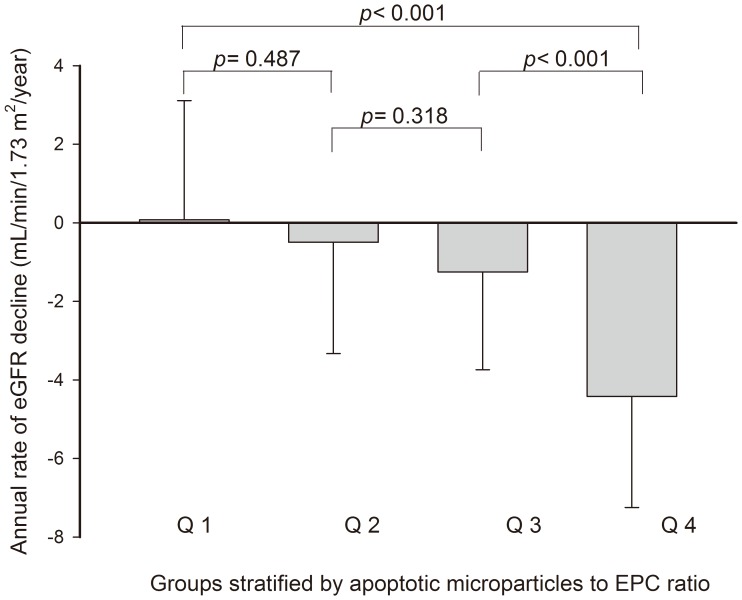
The annual rate of eGFR decline in the study groups stratified by EMP/EPC ratio. All patients were divided into 4 groups according to EMP/EPC ratio in quartiles: group 1 (Q1), with EMP/EPC ratio ≤0.5; group 2 (Q2), with EMP/EPC ratio >0.5 and ≤2.0; group 3 (Q3), with EMP/EPC ratio >2.0 and ≤11.7; group 4 (Q4), with EMP/EPC ratio >11.7. As seen, there was a significantly positive association between the EMP/EPC ratio and the subsequent decline in eGFR.

As illustrated in Figure 2, we graphically reported the values of the annual rate of decline of eGFR in the study groups stratified by EMP/EPC ratio. There was a significant positive association between the EMP/EPC ratio and the rate of decline in eGFR (mean ΔeGFR/y, Q1 vs. Q2 vs. Q3 vs. Q4 = 0.08±3.04 vs. −0.50±2.84 vs. −1.25±2.49 vs. −4.42±2.82 mL/min/1.73 m^2^, *p*<0.001).

**Figure 3:**
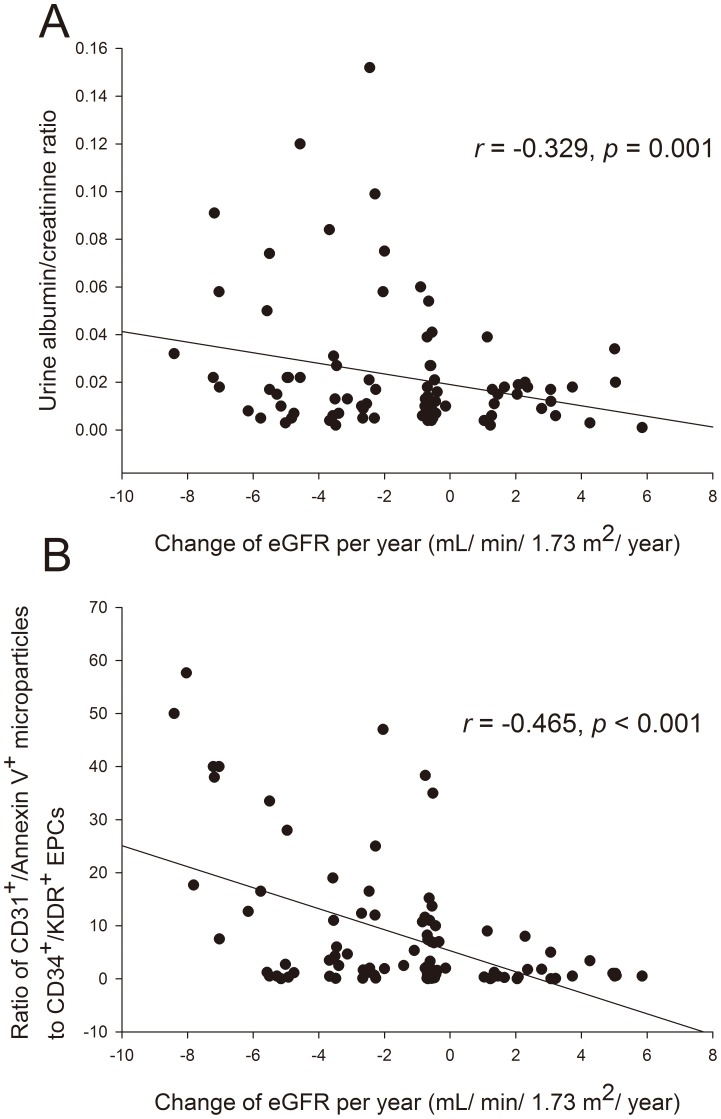
The association between microalbuminuria, EMP/EPC ratio, and eGFR decline rate. The distribution and association between: (A) the microalbuminuria (urine albumin/creatinine ratio) and annual change of estimated glomerular filtration rate (eGFR); and (B) the ratio of CD31+/Annexin V+ microparticles to CD34^+^KDR^+^CD133^+^ endothelial progenitor cells (EPCs) and annual change of eGFR in 100 hypertensive patients.

To further clarify the association between change in eGFR and EMP/EPC ratio after adjustment for other risk factors, we performed simple and multiple regression analyses that included demographic variables and cardiovascular risk factors. As demonstrated in Figure 3, the mean annual decline in eGFR was significantly correlated with circulating EMP to EPC ratio (*r* = −0.465, *p*<0.001) and urine albumin/creatinine ratio (*r* = −0.329, *p* = 0.001). Moreover, circulating EMP concentration, EPC level alone, and Framingham risk score showed a marginal association with eGFR decline (*p* value around 0.05). Besides, the correlations between the EMP/EPC ratio and the annual decline rate in eGFR in patients with CKD were consistent with the result in the overall population (eGFR <60, *r* = −0.490, *p* = 0.015).

By multivariate analysis, EMP to EPC ratio, microalbuminuria, and the Framingham risk score were significant independent factors to subsequent decline in eGFR (Table 3). The combination of EMP and EPC levels (EMP/EPC ratio) conferred the most important associated factor of renal function decline in this model (*r* = −0.340, *p* = 0.003). Additionally, a marginal association between the EMP/EPC ratio and the urine albumin/creatinine ratio was noted (*r* = 0.328, *p* = 0.069).

**Table 3:** Univariable and Multivariable Associations with Annual Change in eGFR Calculated by CKD-EPI Equation.

	Univariate analysis		Multivariate analysis*	
	Coefficient	*p* value	Coefficient	*p* value
EMP/EPC ratio	−0.465	<0.001	−0.340	0.003
EMP	−0.308	0.045	–	–
EPC	0.192	0.055	–	–
Microalbuminuria (ACR)	−0.329	0.001	−0.226	0.015
Framingham risk score (%)	−0.245	0.043	−0.203	0.038
Baseline serum creatinine	−0.138	0.167	0.134	0.244
Uric acid	0.109	0.277		
Fasting blood glucose	0.089	0.372		
Antihypertensive therapy duration (years)	0.082	0.413		
LDL	−0.050	0.620		
Hs-CRP	0.041	0.683		
NT-pro BNP	0.041	0.688		

* The multivariate regression model included all available variables with p value <0.200 (except EMP and EPC levels alone).

eGFR, estimated glomerular filtration rate; CKD-EPI equation, Chronic Kidney Disease Epidemiology equation; EMP/EPC ratio, Ratio of CD31+/annexin V+ endothelial apoptotic microparticles (n/µL) to CD34^+^KDR^+^CD133^+^ endothelial progenitor cell (n/mL); ACR, albumin/creatinine ratio; Framingham risk score included parameters with age, gender, total cholesterol, high-density lipoprotein, smoking, and systolic blood pressure; LDL, low-density lipoprotein; hs-CRP, high-sensitivity C-reactive protein; NT-pro-BNP, N terminal pro- brain natriuretic peptide (pg/mL).

## Discussion

To the best of our knowledge, this is the first study to show that increased EMP to EPC ratio in hypertensive patients is associated with subsequent decline in GFR. Even when the relationship between the EMP/EPC ratio and eGFR is expressed by a moderate correlation coefficient, our data suggest that the association is as clinically important as that documented by the microalbuminuria and Framingham risk score. These findings demonstrate that the reduced vascular repair capacity and increased endothelial damage indicated by higher EMP to EPC ratios may exacerbate the decline in renal function in patients with hypertension, and that therapies to improve circulating EPC levels and reduce endothelial damage may help to prevent the progression of hypertensive kidney disease.

Chronic kidney disease (CKD) is a major public health problem [33]. Renal insufficiency has been recognized as an independent risk factor for all-cause mortality [1], [2] as well as adverse CVD outcomes including myocardial infarction, stroke, and progression of heart failure [2]–[4], [7]. This has been demonstrated in several groups, particularly in elderly individuals and in the general population [33], and also in selected patients with hypertension, diabetes mellitus, myocardial infarction, or congestive heart failure [5]–[7]. Therefore, the detection of renal insufficiency should alert practitioners to routinely assess renal function to correctly define the total burden of cardiovascular diseases. Because clinical studies indicate that declining renal function is associated with an increased risk for cardiovascular and all-cause mortality, the identification of hypertensive patients at high risk for developing CKD is an important issue for the primary and secondary prevention of CKD in the community.

There are various etiologies thought to contribute to the pathogenesis of atherosclerosis in patients with CKD, including oxidative stress, inflammation, and endothelial dysfunction. It has become clear that the vascular endothelium is a highly active organ that affects vascular tone, platelet aggregation, monocyte adhesion, and smooth muscle cell proliferation [8]. The integrity and functional activity of the endothelial monolayer have been shown to play critical roles in atherogenesis [9]. Extensive endothelial cell damage can result in endothelial cell apoptosis, with subsequent loss of integrity of the endothelium. The extent of endothelial damage may represent a balance between the magnitude of injury and the capacity for repair and can predict cardiovascular event rates [30], [34], [35]. Recent evidence suggests that the injured endothelial monolayer is regenerated partly by bone marrow-derived EPCs, and the circulating EPC level has been shown to be associated with endothelial function [13]. A reduced level or function of EPCs in the peripheral circulation independently predicts atherosclerotic disease progression and future cardiovascular events [14]–[18], which suggest a pivotal role for endogenous vascular repair by EPCs in modulating the clinical course of CAD.

Microparticles carry membrane proteins and phospholipids of the parent cell (e.g. CD31 when derived from endothelial cells), and can also be derived from leukocytes, erythrocytes, or platelets [19]. The current knowledge on microparticle formation derives mainly from experiments on isolated or cultured cells showing that both cell activation and cell apoptosis can lead to microparticle release. Increasing evidence has indicated that levels of circulating EMPs are elevated as a consequence of vascular endothelial cell damage [19], [23], and that these microparticles may be proatherogenic [20]–[22]. Our recent data showed that hypertensive patients with micro- or macroalbuminuria have increased EMP levels and decreased numbers of circulating EPCs [24]. Elevated levels of EMPs and reduced numbers of circulating EPCs may play a synergistic role in the process of atherosclerosis in patients with hypertension and may additively predict cardiovascular outcomes. These findings may explain the pathogenetic processes that link microalbuminuria to endothelial repair capacity and the endothelial injury index, implying that hypertensive nephropathy could be a cause or a consequence of endothelial dysfunction. Of interest, clinical evidence has indicated that endothelial dysfunction is already present in the early stages of renal insufficiency [11]. Impaired endothelial function is associated with a greater decline in renal function even after adjustment for the known cardiovascular risk factors [12]. However, no previous study has mentioned the possible role of circulating EMP and EPC levels in the progression of renal disease in hypertensive patients. In this study, we have extended previous results and further demonstrated that an increased EMP/EPC ratio in hypertensive patients is associated with subsequent declines in eGFR. These findings suggest that reduced vascular repair capacity and increased endothelial damage, assessed by the EMP/EPC ratio, may play a pivotal role in renal function decline in hypertensive patients.

However, there has been some controversy regarding whether EMPs are just the sequela of vascular injury or have a pathological role. Previous study in patients with end-stage kidney disease suggested that EMPs may play a pro-atherogenic role other than being a consequence of vascular damage [23]. Although the cellular mechanism of action of EMPs largely remains unclear, our recent study also showed that EMPs isolated from hypertensive patients with nephropathy may have impaired EPC function, increased reactive oxygen species production, and enhanced cellular apoptosis and senescence [24]. These results are in line with previous report showing that the circulating EMPs derived from patients with myocardial infarction had proinflammatory as well as procoagulant effects and impaired the release of nitric oxide from endothelial cells [36]. Increased ratio of the levels of circulating CD31+/annexin V+ EMPs to EPCs is associated with subsequent declines in GFR in hypertensive patients, and levels of circulating CD31+/Annexin V+ EMPs significantly predict the degree of urinary albumin excretion rate in humans with hypertension [24], suggesting a vicious cycle of microparticles on injured endothelium in hypertensive patients with nephropathy. These findings may partly explain the pathogenetic processes that link EPC, EMP and renal disease.

On the other hand, EMPs also stimulate angiogenesis and differentiation of EPC [37]. Recent studies have shown that EMPs even contain distinct microRNA patterns, suggesting that EMP regulate gene expression of their cells of origin [38]. In the kidney research, Cantallupi *et al.* reported that EMPs from EPCs isolated from healthy subjects protect against acute and long-term consequences of ischemia-reperfusion injury in rats [39]. The findings can be speculated whether circulating EMPs constitute an additional physiological mechanism to counter endothelial damage that may be altered in disease states. It is not certain to what extent these apparently counteracting mechanisms are involved in vivo, and certainly more research is needed in this area.

In this study, levels of hsCRP and NT-proBNP were not sufficient to predict short-term changes in renal function as significant independent factors. This suggested that the changes in the EMP/EPC ratio might be more sensitive than these biomarkers for predicting the progress of hypertensive kidney disease. Although not tested in the present study, the prevention of progressive renal disease in hypertensive patients may rely not only on rigorous blood pressure control, but also on other measures known to improve endothelial function and reduce endothelial damage by enhancement of circulating EPC levels.

### Study Limitation

Some limitations of this study should be mentioned. First, the sample size is rather small, and was assembled from a single center. Therefore, further larger confirmative studies are needed to verify the current result. Second, no consensus or standardized assay for the measurements of EMP and EPC levels has been established for routine clinical testing, and the number of this study population was not estimated by power calculation. Further, although we used the newer equation from CKD-EPI for GFR estimation, which has demonstrated less bias at higher GFR values and has been validated in Asian and Chinese populations, we did not have a direct measurement of GFR in this study. At last, we cannot exclude the possibility that the EMP level and EMP/EPC ratio increased as the consequence of impaired kidney function and reduced EMP clearance. However, the impact of this clearance effect is difficult to be measured precisely because of lacking the reference data of EMP clearance in deferent stages of CKD according to the best of our knowledge.

### Conclusions

In conclusion, this is the first study to show that an increased ratio of the levels of circulating CD31^+^/annexin V^+^ EMPs to EPCs is associated with subsequent declines in GFR in hypertensive patients. The results suggest that reduced vascular repair capacity and escalating endothelial damage may contribute to deterioration of renal function in hypertensive patients. These findings may explain the pathogenetic processes coupling the balance of endothelial injury and the subsequent pathogenetic progression of hypertensive kidney disease.
